# Selective inhibition of interleukin 6 receptor decreased inflammatory cytokines and increased proteases in an experimental model of critical calvarial defect

**DOI:** 10.1590/1414-431X2024e13913

**Published:** 2024-08-19

**Authors:** R.C.O. Melo, A.A. Martins, G.H.A. Vieira, R.V.S. Andrade, D.N.A. Silva, J. Chalmers, T.M. Silveira, F.Q. Pirih, V.S. Araújo, J.S.P. Silva, M.L.D.S. Lopes, R.F.C. Leitão, R.F. Araújo, I.L.G. Silva, L.J.T. Silva, E.G. Barbosa, A.A. Araújo

**Affiliations:** 1 Departamento de Odontologia, Universidade Federal do Rio Grande do Norte, Natal, RN, Brasil; 2 Section of Periodontics, School of Dentistry, University of California, Los Angeles, CA, USA; 3 Departamento de Morfologia, Universidade Federal do Ceará, Fortaleza, CE, Brasil; 4 Departamento de Morfologia, Universidade Federal do Rio Grande do Norte, Natal, RN, Brasil; 5 Departamento de Ciências Farmacêuticas, Universidade Federal do Rio Grande do Norte, Natal, RN, Brasil; 6 Departamento de Biofísica e Farmacologia, Universidade Federal do Rio Grande do Norte, Natal, RN, Brasil

**Keywords:** Inflammation, Bone, Tocilizumab, Critical defect, Cytokines

## Abstract

Considering the lack of consensus related to the impact of selective IL-6 receptor inhibition on bone remodeling and the scarcity of reports, especially on large bone defects, this study proposed to evaluate the biological impact of the selective inhibitor of interleukin-6 receptor (tocilizumab) in an experimental model of critical calvarial defect in rats. In this preclinical and *in vivo* study, 24 male Wistar rats were randomly divided into two groups (n=12/group): defect treated with collagen sponge (CG) and defect treated with collagen sponge associated with 2 mg/kg tocilizumab (TCZ). The defect in the parietal bone was created using an 8-mm diameter trephine drill. After 90 days, the animals were euthanized, and tissue samples (skull caps) were evaluated through micro-CT, histological, immunohistochemistry, cytokines, and RT-qPCR analyses. Tocilizumab reduced mononuclear inflammatory infiltration (P<0.05) and tumor necrosis factor (TNF)-α levels (P<0.01) and down-regulated tissue gene expression of BMP-2 (P<0.001), RUNX-2 (P<0.05), and interleukin (IL)-6 (P<0.05). Moreover, it promoted a stronger immunostaining of cathepsin and RANKL (P<0.05). Micro-CT and histological analyses revealed no impact on general bone formation (P>0.05). The bone cells (osteoblasts, osteoclasts, and osteocytes) in the defect area were similar in both groups (P>0.05). Tocilizumab reduced inflammatory cytokines, decreased osteogenic protein, and increased proteases in a critical bone defect in rats. Ninety days after the local application of tocilizumab in the cranial defect, we did not find a significant formation of bone tissue compared with a collagen sponge.

## Introduction

Bone healing is a complex process with closely linked phases of inflammation, regeneration, and remodeling (1). Among mediators involved in this dynamic activity, interleukin-6 (IL-6) is considered one of the most prominent pro-inflammatory cytokines. It has various activities in multiple cell types, including those in bone tissue (2). The role of IL-6 in physiological bone turnover has been extensively investigated, demonstrating that it can modulate osteoblast and osteoclast activity (3).

In this context, it is well-known that healing complications occur more frequently in patients with inflammatory disorders, which are often associated with increased circulating pro-inflammatory cytokine levels (including IL-6), such as rheumatoid arthritis (4). Patients with rheumatoid arthritis are usually treated with inhibitors of interleukin receptors, and one of the drugs of choice is tocilizumab (5). Formerly known as myeloma receptor antibody, this medicament is a humanized IgG1 IL-6 receptor monoclonal antibody that binds with high affinity to the 80 kDa component of IL-6R (6). This binding subsequently inhibits dimerization of the IL-6/IL-6R complex with membrane-bound gp130, preventing signaling (7).

Kume et al. (8), in a study that evaluated the effect of tocilizumab treatment on bone mineral density of patients with rheumatoid arthritis, revealed an increase in this parameter in those who had osteopenia at baseline. In contrast, Liu et al. (9) showed that the treatment of mesenchymal stem cells with tocilizumab, in addition to not affecting the expression of osteocalcin, inhibited the alkaline phosphatase protein activity, thus demonstrating a negative effect on osteogenesis.

Considering the lack of consensus related to the impact of selective IL-6 receptor inhibition on bone remodeling and the scarcity of reports, especially in large bone defects, the present study aimed to evaluate the role of the tocilizumab on inflammation in an experimental model of critical calvarial defect.

## Material and Methods

### IL-6 R protein docking

The drug tocilizumab was developed to block IL-6 receptors in humans; however, our findings were obtained by conducting biological testing in animal models using *Rattus norvegicus*. To assess the potential for cross-reactivity between tocilizumab and the corresponding IL-6 receptor in rats, computational tests were performed. This involved utilizing the protein sequence of the *Rattus norvegicus* IL-6 receptor obtained from the UniProt server (10). The amino acid sequence was derived through sequence alignment with human IL-6 and the rat protein database, using the BLAST program (11). Structure 8J6F (www.rcsb.org/structure/8J6F) from the protein database was employed for sequence alignment to find the structure most similar to the human protein. The alpha chain of the IL-6 receptor from structure 8J6F was used for this purpose. The IL-6 receptor structure lacks experimentally determined structures that could be used to dock the tocilizumab drug. Therefore, homology modeling was conducted using the I-TASSER (12) server to obtain such a structure. Subsequently, homology models for the human and rat IL-6 receptor structures were docked to tocilizumab using the Cluspro server (13). Interaction scores were evaluated to classify the mode of interaction.

### Study design and ethical implications

This is a pre-clinical, *in vivo*, randomized controlled trial. The experimental protocol developed in this research was approved by the animal use ethics committee (CEUA) of the Federal University of Rio Grande do Norte (protocol No. 004/2021).

### Animals and study groups

Twenty-four male Wistar rats (*Rattus novergicus albinus*), provided by the vivarium of the UFRN Biosciences Center, with approximately 200-300 g of body mass and aged 8-12 weeks, were kept under controlled environmental conditions of humidity (45-55%) and temperature (22±2°C), with a 12-h light/dark cycle. The animals had *ad libitum* access to water and commercial feed.

The animals were randomized and distributed into two groups (n=12/group): Control group (CG): 12 animals with critical defects treated with collagen sponge impregnated with 500 µL of saline solution; Tocilizumab group (TCZ): 12 animals with critical defects treated with collagen sponge impregnated with 500 µL of 2 mg/kg tocilizumab (Actemra^®^, Hoffmann-La Roche Ltd., Switzerland).

For the experimental model described, we required 15 animals in each group and treatment condition to identify statistically significant and biologically relevant differences between baseline performance and the effects of treatment. Specifically, our goal was to collect all data in at least 80% of the rats. We estimated the loss of three rats (death) in each experimental group, given the experimental model used. Starting with 15 animals per group and the treatment condition, we would have data from at least twelve rats (14). Based on these numbers, if treatment caused the sample mean to vary by at least 0.82 standard deviation, we would have an 80% chance of identifying statistical significance. In most cases, a variation of 0.82 standard deviation is quite small, so our design must be efficient in detecting scientifically relevant effects.

### Critical defect in calvaria

The animals were anesthetized through an intraperitoneal injection of 10% ketamine (Vetnil, Brazil) and 2% xylazine (Calmium, Brazil), in doses of 80 and 10 mg/kg, respectively.

**Figure 1:**
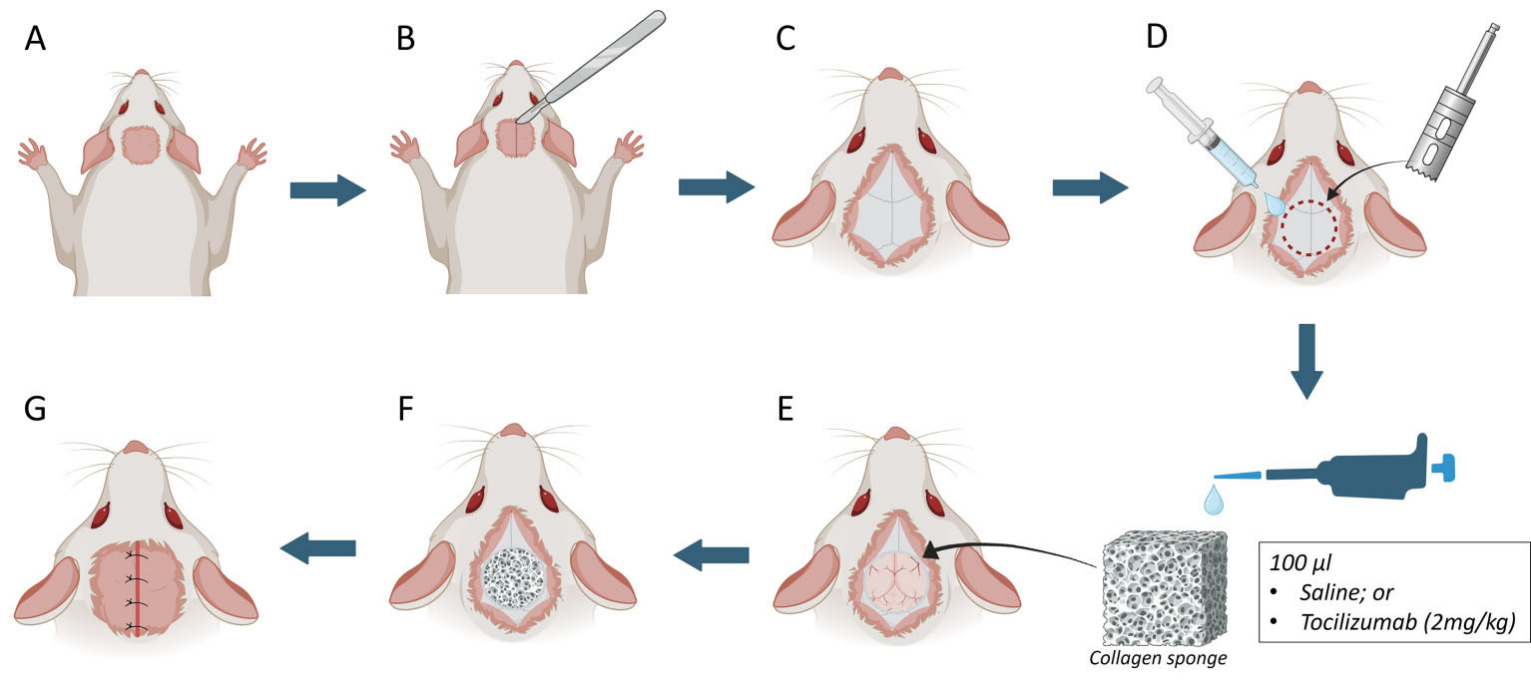
Schematic drawing of the surgical procedure for implantation of collagen sponge with or without tocilizumab. **A**, Trichotomy and asepsis of the surgical area. **B**, Single initial incision in the anteroposterior direction with a scalpel blade for soft tissue disruption. **C**, Exposure of calvaria bone after incision. **D**, A Trephine 8-mm drill was used for drilling the critical bone defect in calvaria under constant irrigation with saline solution. **E**, Addition of 100 µL of saline solution or tocilizumab (2 mg/kg) on the collagen sponge for implantation. **E**, Aspect after implantation of collagen sponge. **F**, Soft tissue approach and simple suture. **G**, Final view of the surgical area.

An skin incision was made from the frontal area of the nose to the external occipital protuberance, and the entire calvarial surface was exposed. Full-thickness flaps, including the skin and periosteum, were elevated, and a single 8-mm cranial defect was created in the center of the parietal bone using an ultra-thin drill driven by a 30,000 rpm electric Micromotor (Surgic XT Plus; NSK, Japan), under constant sterile saline irrigation to prevent bone overheating (14). Then, a collagen sponge (Hemospon^®^, Maquira^®^, Brazil) impregnated with saline or 2 mg/kg tocilizumab (local administration) was placed in the region, filling the defect. The periosteum and the skin were then repositioned and sutured to achieve primary closure, employing a 4-0 surgical suture thread (Ethicon, Johnson & Johnson, Brazil). The surgical experimental process is shown in Figure 1.

For postoperative care, 5 mg/kg tramadol was administered intraperitoneally for five days (12/12 h), and 0.1 mg/mL tramadol was associated to the drinking water (*ad libitum*) for the same time period (15).

### Euthanasia

The animals were euthanized 90 days after the surgery and local treatment, using ketamine 10% (Vetnil) and xylazine 2% (Calmium), in doses of 240 and 30 mg/kg, respectively. Calvariae tissues were collected and used for analysis.

### Micro-CT analysis

Calvariae (n=4 samples/group) were dissected and fixed with 4% paraformaldehyde in 0.1 M phosphate-buffered saline for 24 h. The samples were scanned using a high-resolution micro-CT (SkyScan 1275, Sky-Scan N.V., Belgium) with an image resolution of 15 µm, an X-ray source of 55 kV and 167 µA, and an aluminum filter of 0.5 mm. Image datasets were reconstructed using the NRecon (SkyScan N.V.) program version 1.7.4.6, with ring artifact correction of 5, beam hardening correction of 20%, and fine tuning. After reconstruction, the images were viewed and reoriented in the transaxial, coronal, and sagittal planes with the DataViewer software (SkyScan N.V.). Volumetric analysis was performed using CTAn software (SkyScan N.V., version 1.13.11.0). The regions of interest (ROI) were standardized in 40 sections per sample, starting at the highest defect edge identified by the coronal view. From the transaxial view, each selected section received an individual delimitation with the same pattern, covering the entire area of the defect and extending 3.0 mm beyond the edge of the defect. Binary selection of a dataset with automatic threshold values, morphometry, and 3D analysis were performed. Bone volume (BV) and tissue volume (TV) were measured to calculate percentage bone volume (BV/TV). In addition, porosity percentage, trabecular separation, trabecular number, and trabecular thickness were assessed.

### Histological analysis (HE)

Cranial specimens (n=4 samples/group) were fixed in 10% formalin for 24 h and submerged in 10% thylenediamine tetraacetic acid solution for 3 months for decalcification. Then, the specimens were dehydrated and impregnated with paraffin; 4-µm-thick sections were obtained from the paraffin blocks, fixed on a slide, and stained with hematoxylin and eosin. An experienced pathologist independently performed histological analyses to evaluate inflammation, bone cells, and bone repair. These assessments were made blindly, utilizing a conventional optical microscope (Olympus^®^ CH2, Olympus Optical Co. Ltd, Japan). The scoring system used to evaluate inflammation and bone formation was: inflammation (14): score 0 - absence of inflammatory cells; score 1 - weak presence of inflammatory cells; score 2 - moderate presence of inflammatory cells; and score 3 - intense presence of inflammatory cells. Bone tissue formation (16): score 1 - formation of new tissue (filling of the defect with connective tissue, which contains blood capillaries, fibroblasts, macrophages, and newly formed collagen fibers); score 2 - dense connective tissue, suggesting the differentiation of bone tissue with the presence of a large number of osteogenic and osteoprogenitor cells with fiber organization; score 3 - presence of new bone in which connective tissue differentiates to form or indicate a bone matrix; and score 4 - formation of mature bone tissue.

The histological analysis also included identifying whether bone cell types - osteoblasts, osteocytes, and osteoclasts - were present or absent.

### Immunohistochemical analysis

The samples from HE analysis (n=4 samples/group) were used for immunohistochemistry analysis. Histological sections of 3-μm thickness from paraffin blocks were placed on glass slides previously prepared with adhesives based on 3-aminopropyltriethoxy silane (Sigma Chemical Co., USA). The slides were subjected to the LSAB immunoperoxidase method using the following primary anti-human antibodies: anti-osteopontin (1:400) and anti-osteocalcin (1:400), anti-MMP-9 (1:400), anti-RANKL (1:400), and anti-cathepsyn (1:400) (Santa Cruz Biotechnology, USA). Immunoreactivity was visualized using a colorimetric detection kit following the protocol provided by the manufacturer (TrekAvidin-HRP Label + Kit from Biocare Medical, USA), and the following scores were assigned according to the percentage of cell marking (14): Score 0: absence of immunostaining; Score 1: weak staining (<25% positive cells); Score 2: moderate staining (25 to 50% positive cells); and Score 3: intense immunostaining (>50% positive cells).

### Cytokines by ELISA immunoassay (TNF-**α** and IL-1**β**)

The periosteum corresponding to the surgical area was collected and divided for cytokine analysis (n=4/group) and RT-qPCR analysis (n=4/group). Segments of periosteum obtained from calvarial specimens (n=4/group) were preserved at -80°C following euthanasia. These samples were homogenized in phosphate buffered saline (PBS) using a rotary homogenizer for sample and tissue processing (17). IL-1β and tumor necrosis factor (TNF)-α levels were determined using a commercial ELISA kit (R&D Systems, USA). Briefly, microtiter plates were coated with antibodies against IL-1β and TNF-α overnight at 4°C. After blocking the plates, the samples and standard at various dilutions were added in duplicate and incubated at 4°C for 24 h. After washing the plates (three times with buffer), biotinylated polyclonal anti-TNF-α, diluted 1:1000 with assay buffer 1% BSA, was added to the wells. After further incubation at room temperature for 1 h, the plates were washed and streptavidin-HRP, diluted 1:5000, was added to each well. The chromogenic reagent O-phenylenediamine was added 15 min later and the plates were incubated in the dark for 15 min. The enzymatic reaction was interrupted with H_2_SO_4_, and the absorbance was measured at 490 nm using UV-VIS spectrophotometry. The results are reported in pg/mL (18).

### RT-qPCR

The periosteum corresponding to the surgical area was collected and divided for cytokine analysis (n=4/group) and RT-qPCR analysis (n=4/group). Ribonucleic acid (RNA) was isolated from the periosteum using Trizol reagent (Invitrogen, USA), following the manufacturer's instructions. RNA was quantified by NanoDrop (Thermo Fisher Scientific Inc., USA), and the purity of the samples was verified by 260/280 ratios >1.8. Five micrograms of isolated total RNA (10 μL) were transcribed to cDNA in a reaction mixture containing 2 μL 10× RT buffer, 0.8 μL 25× dNTP Mix, 2 μL 10× RT oligo dT, 1 μL MultiScribe reverse transcriptase, and 4.2 μL H_2_O (High-Capacity cDNA Reverse Transcription Kit, USA) in a total volume of 20 μL. The reaction mixture was incubated at 25°C for 10 min, 37°C for 120 min, 85°C for 5 min, and 4°C for 120 min.

The cDNA was stored at −80°C until further use. qPCR was performed using SYBR Green PCR Master Mix (Applied Biosystems by Life Technologies, USA), according to the manufacturer's instructions. The sequences of the primers are listed in Table 1. To compare gene expression under different conditions, the expression under each condition (normalized to ACTB, the endogenous control) was quantified relative to the control condition. qPCR amplification was performed in a CFX Connect system (Bio-Rad Laboratories, SA) under the following conditions: 50°C for 2 min and 95°C for 10 min, followed by 40 cycles of 95°C for 15 s and 60°C for 60 s. The relative expression levels of the genes were calculated using the threshold cycle (2^−ΔΔCT^) method (19).

**Table 1:** Primer sequences of genes used in the study.

Primer	Sequence
*β-actin*	
Forward	AGGCCAACCTGTAAAAGATG
Reverse	TGTGGTACGAGAGGCATAC
*IL-6*	
Forward	CTTCCAGCCAGTTGCCTTCTTG
Reverse	TGGTCTGTTCTGGGTGGTATCC
*BMP-2*	
Forward	GGGACCCGCTGTCTTCTAGT
Reverse	TCAACTCAAATTCGCTGAGGAC
*RUNX-2*	
Forward	CCTCTGACTTCTGCCTCTGG
Reverse	TAAAGGTGGCTGGGTAGTGC

*IL-6*: Interleukin 6; *BMP-2*: Bone morphogenetic protein 2; *RUNX-2*: RUNX family transcription factor 2.

### Statistical analysis

The results obtained were submitted to descriptive and inferential statistics in the software Prism 8.0 program (GraphPad, USA). The animal was considered the experimental unit. Data were analyzed for normality distribution (Shapiro-Wilk test), and independent samples *t*-test or Mann-Whitney test was applied accordingly. For all analyses, a significance level of 5% (P<0.05) was considered.

## Results

### IL-6 R protein docking

**Figure 2:**
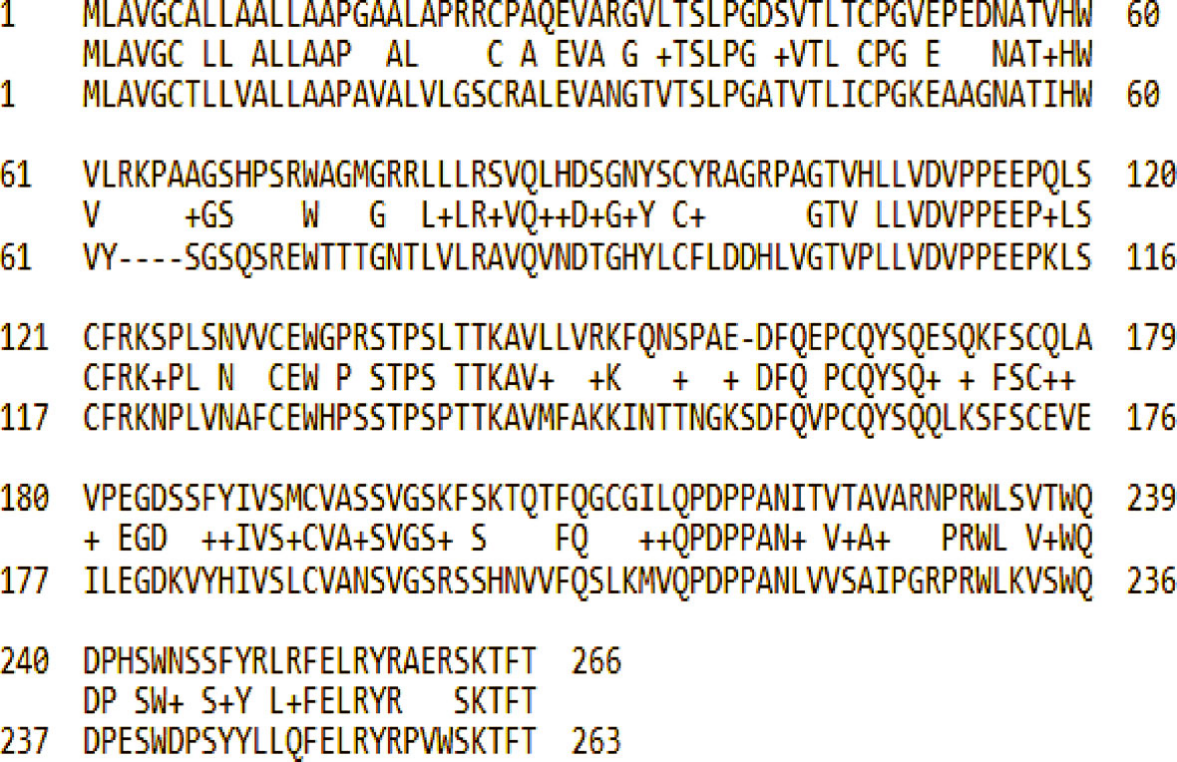
Interleukin (IL-6) receptor sequence correspondence. Interleukin-6 receptor subunit alpha isoform X3" (Sequence ID: XP_006232650.1).

**Figure 3:**
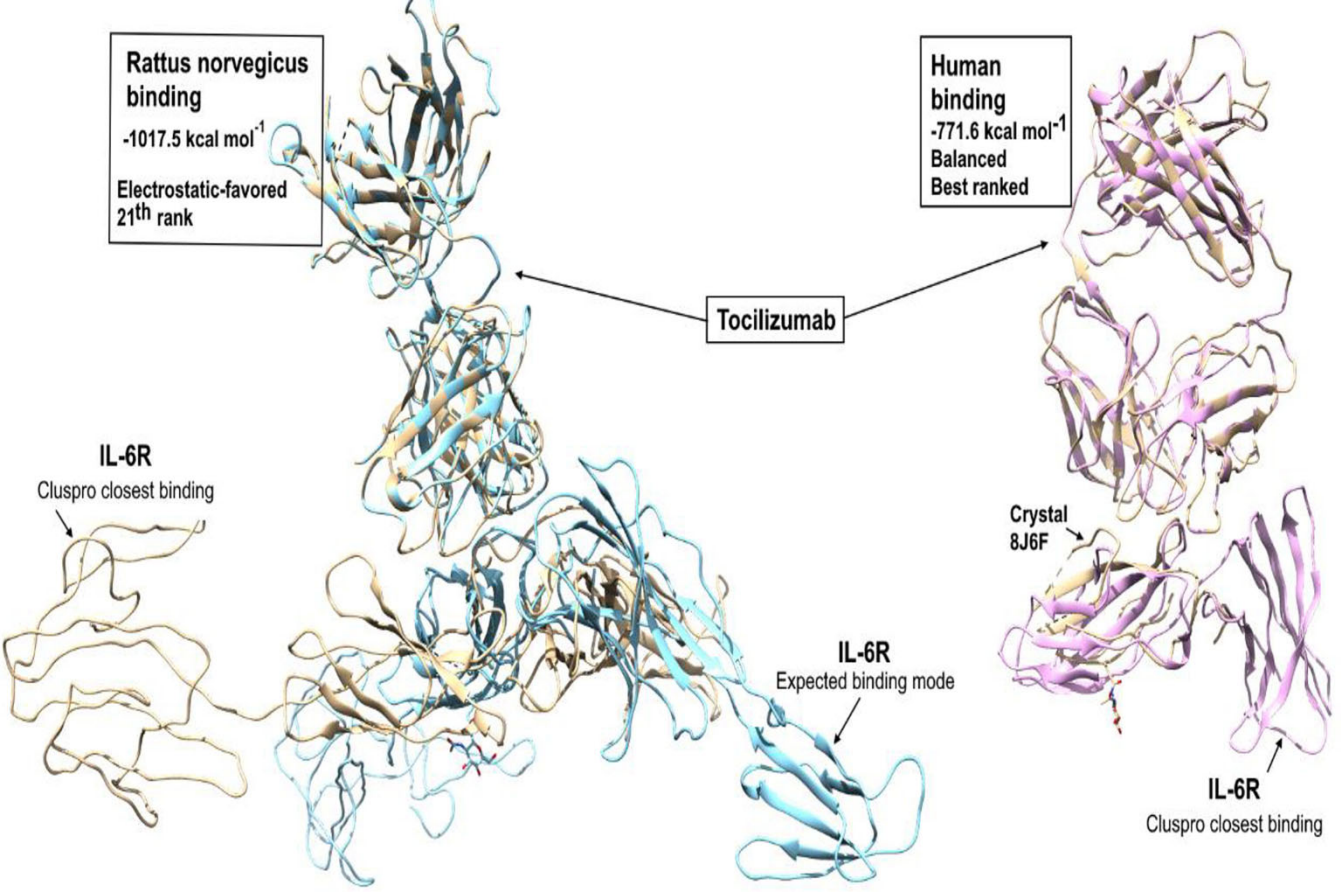
*In silico* analysis. Comparison of the Cluspro docked interaction for the rat interleukin (IL)-6 receptor with the best approximated interaction for human IL-6R binding (expected binding mode, shown in blue). The human docked structure exhibits a remarkable similarity between the experimental structure (shown in pink). It is important to note that while the score values are more negative for the rat IL-6, direct comparison is not feasible due to differences in structure size and scoring methodology. In the case of human structure docking, a balanced Cluspro score was sufficient to reproduce the experimental structure of 8J6F. Conversely, for the rat structure, scoring based on electrostatic-favored parameters was necessary, resulting in inherently more negative energy values. Consequently, direct comparison of score values was not viable; rather, their relative positions in the ranking were considered.

Sequence alignment revealed a corresponding IL-6 receptor in rats with 57% identity and 70% positively aligned residues. The sequence corresponded to the “interleukin-6 receptor subunit alpha isoform X3” (Sequence ID: XP_006232650.1, Figure 2). Sequence alignment demonstrated several alignment gaps, particularly in the tocilizumab recognition region, indicating low similarity in that region. Sequence differences proved to have an impact on the docking results. Only the Cluspro docked structures at the twenty-first position could approach what would be expected for a comparative interaction with the human IL-6 receptor (Figure 3). In contrast to the results obtained for the human interaction, the Cluspro server was able to reproduce a conformation highly similar to the experimentally obtained crystal structure in the top-ranked conformation.

### Outcomes

Two animals from each group died following surgery. Perioperative mortality usually occurs as a result of anesthesia, the surgical procedure itself, or a combination of both factors.

### Micro-CT

**Figure 4:**
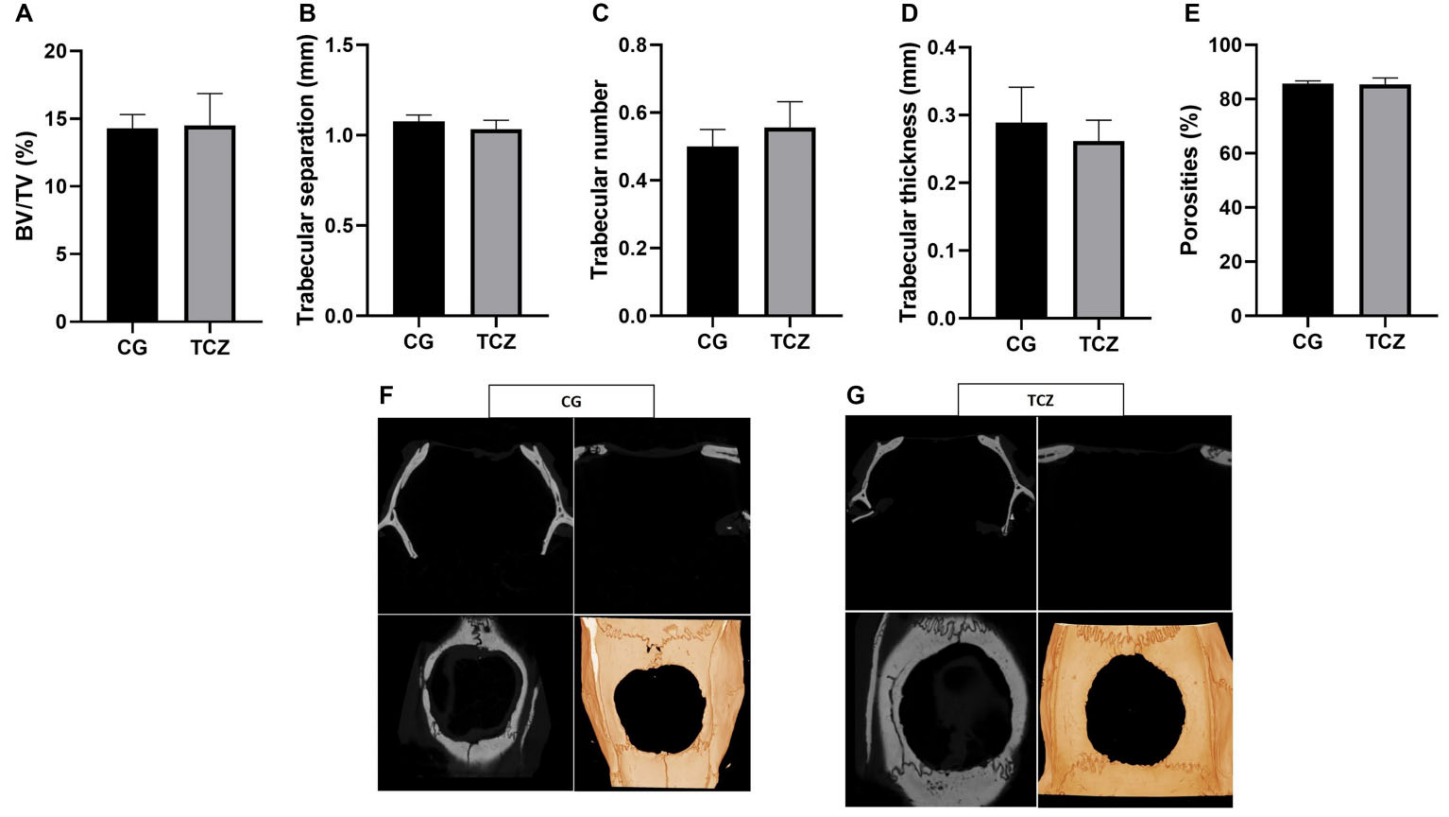
Micro-CT analysis. (**A**) Bone volume (BV)/tissue volume (TV) (%), (**B**) trabecular separation (mm), (**C**) trabecular number, (**D**) trabecular thickness (mm), and (**E**) porosities (%). CG: Control group; TCZ: Tocilizumab group. Data are reported as mean and SD. (P>0.05, Student's *t*-test). Representative 2D and 3D reconstructions of the microtomographic sections for control (**F**) and TCZ (**G**) groups.

Micro-CT analysis revealed that the microarchitecture of the bone tissue margin in terms of BV/TV (%), trabecular separation (mm), number of trabeculae (mm), trabecular thickness (mm), and porosity (%) did not show significant differences between TCZ and control groups (P>0.05; Figure 4).

### Histological analysis

**Figure 5:**
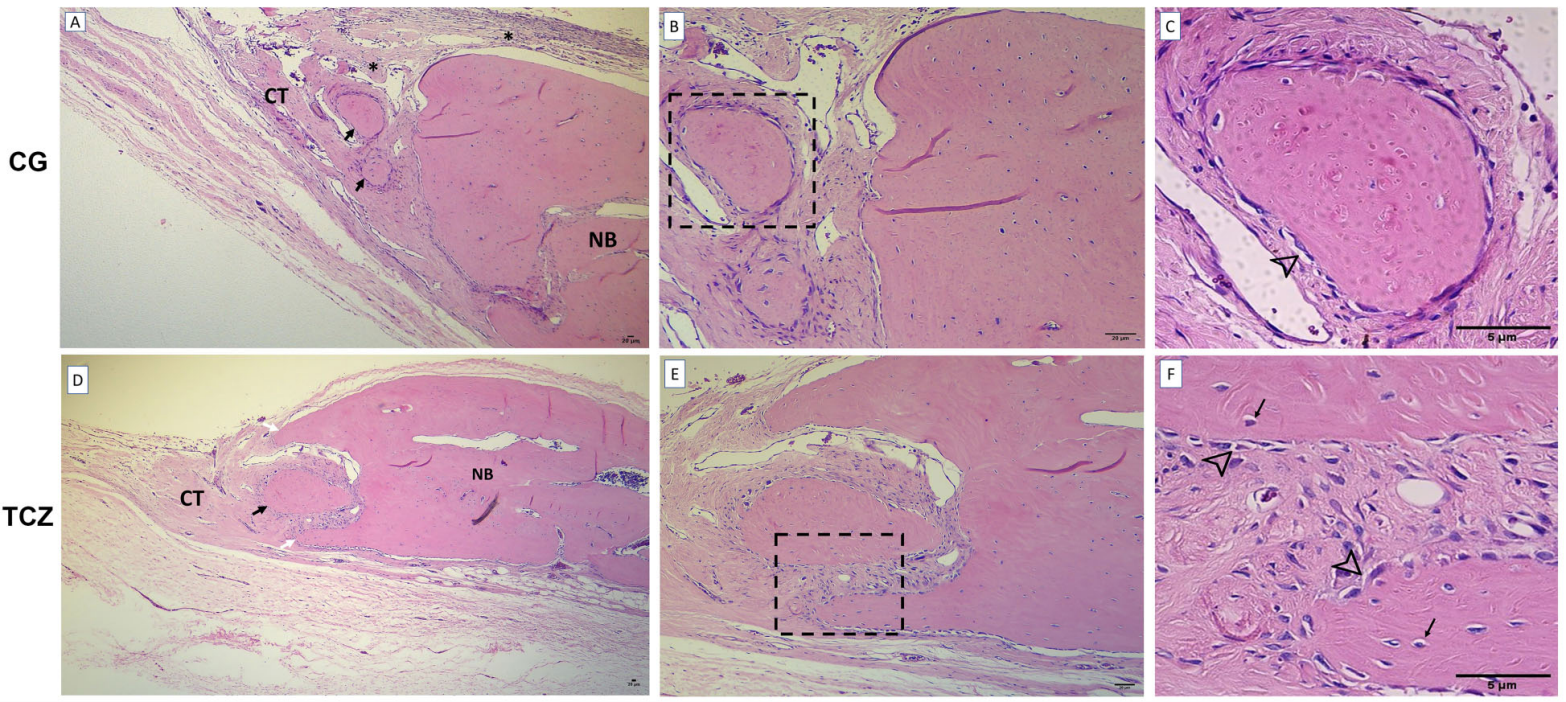
Histological analysis. Representative photomicrographs with H&E staining of the control group (CG, **A**-**C**) and tocilizumab group (TCZ, **D**-**F**). Newly formed bone in the calvarial defect is indicated by large black arrows. Defect margins showing projection-like bone formation are indicated by white arrows. CT: Fibrous connective tissue; NB: native bone. Mild inflammatory infiltrate is indicated by black asterisks. Osteoblastic rimming is indicated by arrowheads. Osteocytes are indicated by thin black arrows. **A**, **B**, **D**, **E**: scale bar 20 μm. **C** and **F**: scale bar 5 μm.

The CG and TCZ groups exhibited new bone formation in the margins of the defect area, while the central area was mainly filled with fibrous connective tissue (Figure 5A-F). No significant differences were observed between the groups regarding the formation and quality of bone (P>0.05; Table 2). The presence of osteocytes, osteoblasts, and osteoclasts was not associated with any group (P>0.05). In both groups, the discreet newly formed bone exhibited osteocytes and osteoblastic cells, while focal osteoclasts were evidenced in only one animal from the CG group and two TCZ animals. Inflammation was absent in the TCZ group, significantly different from CG, which showed a predominantly weak mononuclear inflammatory infiltration (P<0.05; Table 2).

**Table 2:** Inflammation and bone formation histological scores according to the group.

	CG	TCZ	P
Inflammation	1.5 (1-2)	0 (0-0)	0.02
Bone formation	2.5 (2-3)	3 (3-3)	0.42

Data are reported as medians (IQR). CG: Control group; TCZ: Tocilizumab group. Non-parametrical Mann-Whitney test.

### Cytokine assay

**Figure 6:**
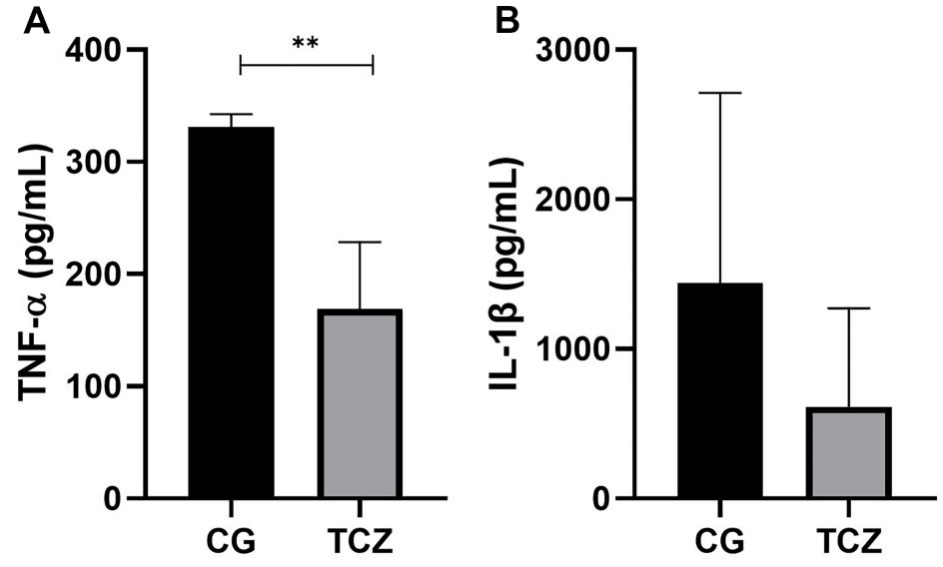
Cytokine assay results of (**A**) tumor necrosis factor (TNF)-α and (**B**) interleukin (IL)-1β tissue levels. CG: Control group; TCZ: Tocilizumab group. Data are reported as mean and SD. **P<0.01 (Student's *t*-test).

Calvaria defects treated with collagen sponge associated to tocilizumab presented lower levels of TNF-α (P<0.01) compared to those treated only with collagen (Figure 6A). IL-1β levels were similar for both groups (Figure 6B).

### RT- qPCR

**Figure 7:**
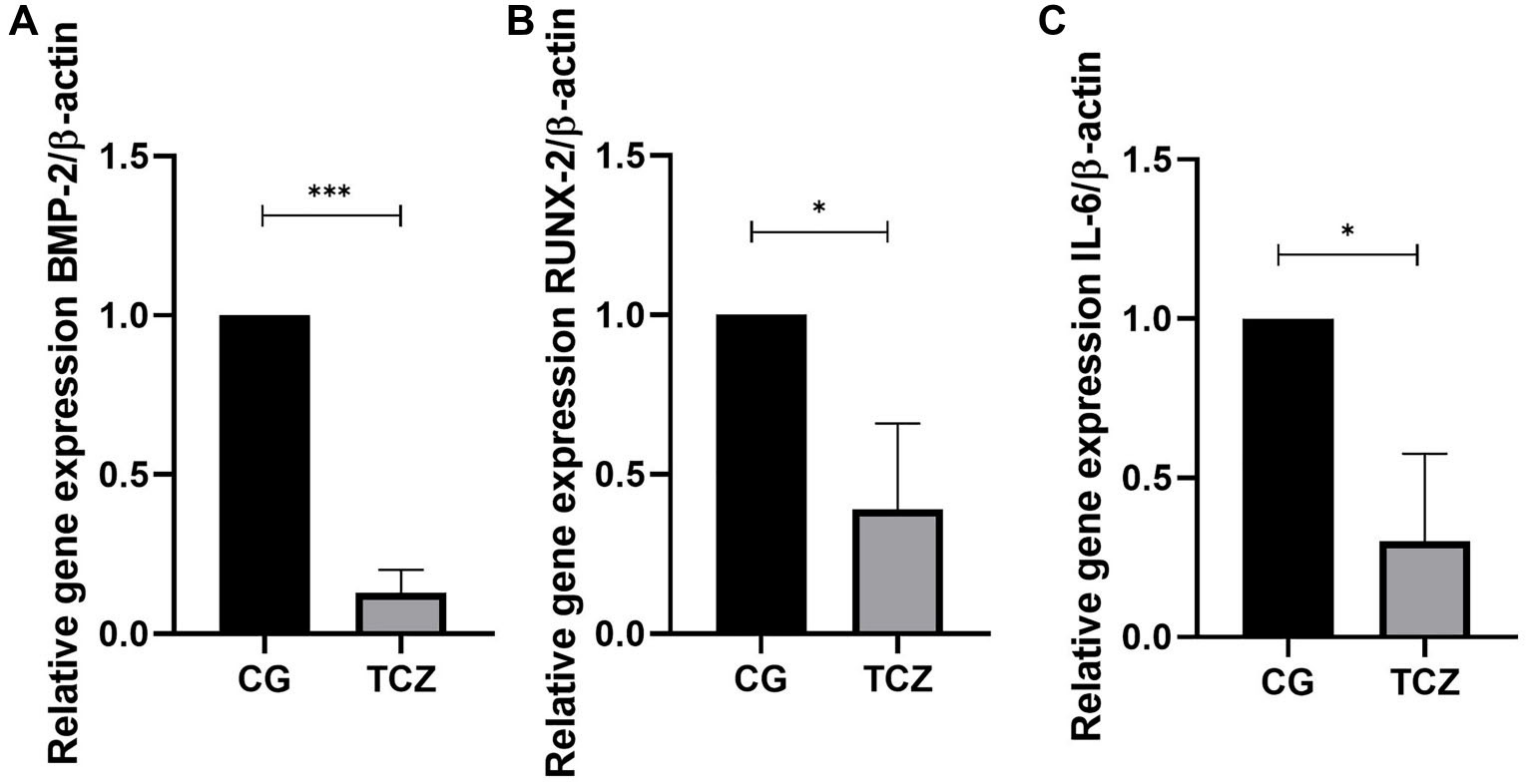
Molecular analysis. (**A**) BMP-2, (**B**) RUNX-2, and (**C**) interleukin (IL-6) relative gene expression measured by RT-qPCR analysis. CG: Control group; TCZ: Tocilizumab group. Data are reported as mean and SD. *P<0.05, ***P<0.001 (Student's *t*-test).

The association of tocilizumab in collagen sponges promoted a down-regulation of the relative expression of *BMP-2* (P<0.001), *RUNX-2* (P<0.05), and *IL-6* (P<0.05) genes (Figure 7A-C).

### Immunohistochemistry

**Figure 8:**
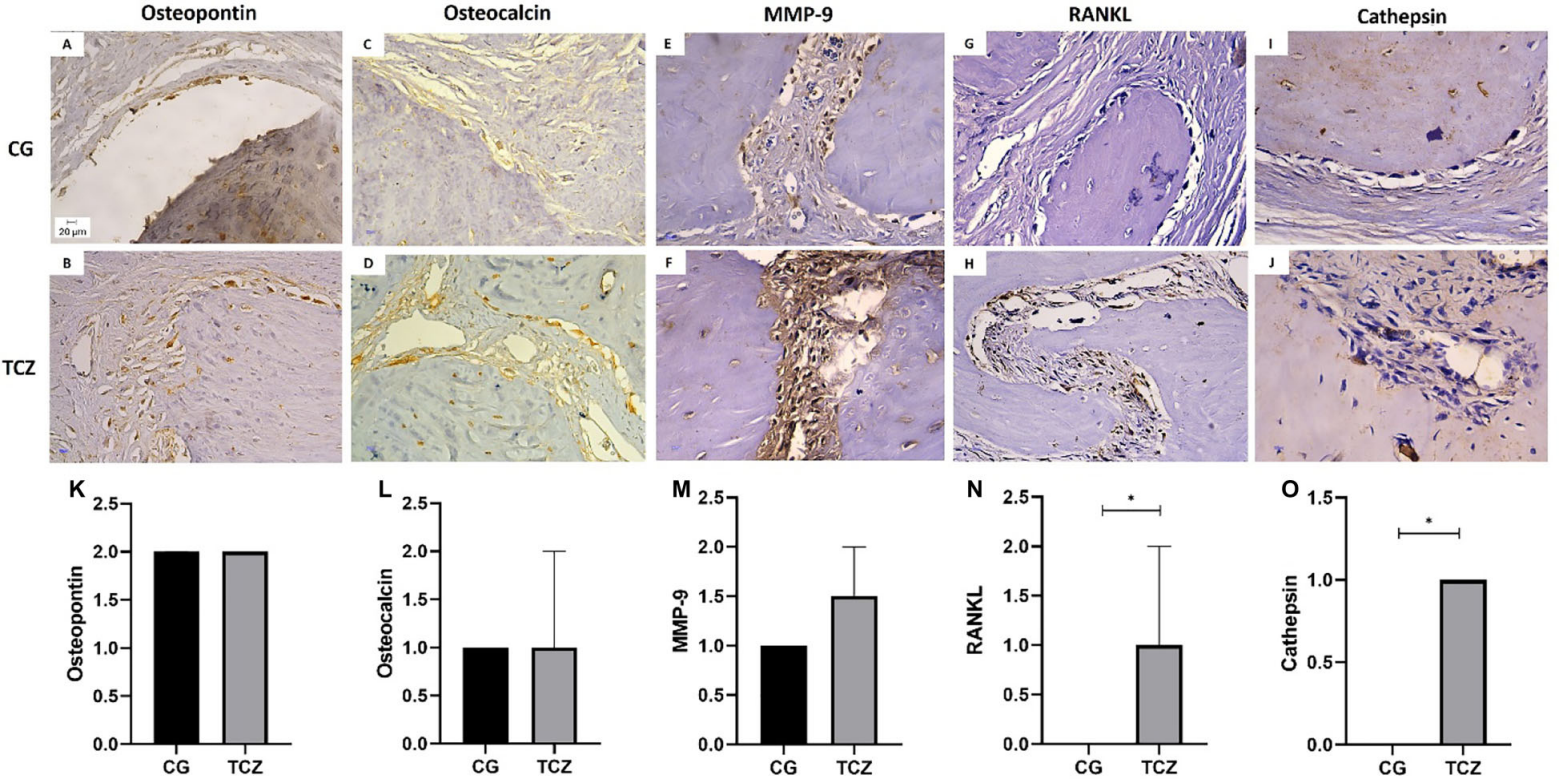
Immunohistochemistry analysis. Photomicrographs of parietal bone in the defect border region showing immunoreactivity to (**A** and **B**) osteopontin, (**C** and **D**) osteocalcin, (**E** and **F**) MMP-9, (**G** and **H**) RANKL, and (**I** and **J**) cathepsin. Scale bar: 20 μm. **K**-**O**, numerical representation of immunohistochemical analysis. CG: Control group; TCZ: Tocilizumab group. Data are reported as medians and IQR. *P<0.05 (non-parametrical Mann-Whitney test).

No significant differences were found between the groups regarding the percentage of cell marking for osteocalcin, osteopontin, and MMP-9 (P>0.05). However, samples from the tocilizumab group showed stronger immunostaining of cathepsin and RANKL than those from the collagen group (P<0.05; Figure 8).

## Discussion

Bone is a dynamic tissue that changes throughout the organism's life, with the capacity for regeneration and repair. However, depending on the size of the defect, the bone tissue does not regenerate completely, making it necessary to carry out bone grafting procedures (20). After fractures, in the context of bone healing, there is a complex process with overlapping phases of inflammation, granulation tissue formation, intramembranous and endochondral ossification, and remodeling by a complex network of inflammatory, angiogenic, osteoanabolic, and osteocatabolic mediators (1,21).

IL-6, a pleiotropic cytokine exerting both pro-inflammatory and anti-inflammatory effects, may play a key regulatory role in the complex fracture-healing cascade (22). Healing complications are reported to occur more frequently in patients with inflammatory disorders, which are often associated with increased circulating IL-6 levels, including osteoporosis, rheumatoid arthritis, and diabetes (4).

Tocilizumab is an IL-6 receptor humanized monoclonal antibody that acts as an IL-6 receptor antagonist (7). Its intravenous and subcutaneous forms are approved in many countries worldwide for treating adults with moderately to severely active rheumatoid arthritis (23). Extensive clinical experience has established this drug's short- and long-term efficacy and safety (24).

Tocilizumab binds to the human interleukin-6 receptor (IL-6R), effectively blocking the signaling of the pro-inflammatory cytokine IL-6. Despite numerous studies in the literature detailing the anti-inflammatory effects of tocilizumab in rats (25-27), a recent study by Lokau et al. revealed that while tocilizumab can indeed inhibit signaling through the human IL-6R, it does not exhibit the same effect in rat cells (28). Our molecular docking studies, aimed at predicting the interactions between tocilizumab in both human and rodent IL-6 receptors, revealed that sequence alignment displayed several gaps, particularly in the recognition region of tocilizumab. This indicates a low similarity between humans and rats in this region. These sequence differences were found to influence docking results. Among all rodent lL6 Cluspro docked structures, one conformation was comparable to the interaction with the human IL-6 receptor. These findings suggested that tocilizumab can inhibit signaling via the rat IL-6R, albeit with less efficacy than its effect on the human IL-6R. In essence, despite the *in silico* analysis indicating a low similarity between the recognition regions of human and rat IL-6 R receptors for tocilizumab antagonism, our *in vivo* findings corroborated previous murine studies demonstrating that this drug mitigates inflammation, including IL-6 levels (25-27). Nevertheless, it is crucial to emphasize that additional validation via proof-of-concept experiments targeting rat IL-6 is essential to confirm tocilizumab's antagonistic effects in rat cells.

We found that tocilizumab could reduce tissue levels of inflammatory markers, such as TNF-α and IL-6, reinforcing the anti-inflammatory effect of this drug. Prystaz et al. (1) demonstrate that the immune response induced by classic IL-6 signaling after fracture is essential for subsequent bone repair. The early short-term blockade of global IL-6 signaling significantly reduced the bone fracture since the global IL-6 inhibition in the early phase after fracture reduced systemic inflammation, the recruitment of immune cells, and bone regeneration (29). Blockade of IL-6R by tocilizumab can result in limited repair in a subset of erosions, particularly in large lesions with sclerosis (30). Liu et al. (9) described that tocilizumab diminished osteogenesis and chondrogenesis of mesenchymal stem cells *in vitro*. On the other hand, tocilizumab reduced inflammation-related bone loss and suppressed tendon inflammation in a juvenile collagen-induced arthritis rat model (27).

Osteoblasts are specialized mesenchymal cells responsible for the process of synthesis and deposition of mineralized and collagen-rich matrix of bone tissue (31). Some proteins produced by this cell type are associated with the deposition of bone matrix, such as osteocalcin and osteopontin (32). Osteocalcin is considered the most abundant bone-specific non-collagenous protein, being associated with the calcium phosphate mineral phase of this tissue (33). Osteopontin (OPN) is a non-collagenous protein involved in biomineralization of bone tissue (34). Our findings showed that selective blockade of the IL-6 receptor wasn't unable to regulate the expression of these markers.

Bone morphogenetic proteins (BMPs) belong to the transforming growth factor beta superfamily and are potent osteogenic agents that stimulate maturation of mesenchymal osteoprogenitor cells into osteoblasts (35). Meanwhile, RUNX2 is a transcription factor responsible for osteoblast differentiation (36). It produces a condensed cell layer of uncommitted mesenchymal cells or osteoblast progenitors by increasing their proliferation and facilitating their differentiation into osteoblast lineage cells (37). Studies have shown that BMPs are necessary for RUNX2 to be active and cooperatively interact to stimulate osteoblast gene expression (38). Our findings showed a significant downregulation of Runx2 and BMP-2 upon administration of tocilizumab. Considering that BMP-2s are up-regulated in rheumatoid arthitis patients in response to pro-inflammatory cytokines (39), the attenuated inflammation promoted by tocilizumab administration may reflect the reduction of these markers.

In addition, immunohistochemistry analysis revealed that tocilizumab increased RANKL and cathepsin immunostaining. The expansion of cortical bone is related to endosteal bone loss through biological processes that further depend on mechanical stimuli. High levels of RANKL can promote severe bone loss and a limited increase in periosteal bone formation. A concomitant increase of cathepsin K triggers periostin degradation, limiting the compensatory stimulation of modeling-based bone formation (40).

Although tocilizumab reduced proinflammatory markers and osteogenic proteins, beyond increasing proteases, no significant impact was found in the quality of bone tissue in the defect area, as revealed by micro-CT evaluation. This may have occurred due to the type of the defect (critical size) and the inability of the collagen sponge to induce bone formation and maturation during the experiment period (90 days). Reinforcing this hypothesis, the histologic data showed that the defect area presented a dense connective tissue in both groups, which suggested that the early phase (differentiation) of bone tissue structuring was occurring. The use of a single 90-day sacrifice may explain the absence of differences in certain parameters; when assessed over a longer period, these molecular findings could show a greater impact on the new bone structure. Also, if animals were sacrificed at shorter time intervals (15, 30, and 60 days), we could have assessed even earlier changes in the bone repair process.

We used tocilizumab to better understand the local effect of this medicine on bone defects. Tocilizumab reduced significantly IL-6 and TNF-α. At 90 days after local application of tocilizumab to a cranial defect, tocilizumab did not show improved formation of bone compared with collagen sponge. Lower levels of proteins and transcription factors involved in osteogenesis, such as BMP-2 and RUNX2, and increased proteases, such as RANKL and cathepsin, were found.
